# The complete mitochondrial genome of the yellowfin shiner, *Notropis lutipinnis*

**DOI:** 10.1080/23802359.2020.1809541

**Published:** 2020-08-26

**Authors:** Karen E. Bobier

**Affiliations:** Department of Genetics, University of Georgia, Athens, Georgia

**Keywords:** Mitochondrial genome, *Notropis lutipinnis*, phylogenetic analysis

## Abstract

The complete mitochondrial genome of the yellowfin shiner (*Notropis lutipinnis*) 16,706 bp and contained 13 protein coding genes, 2 rRNAs, 22tRNAs, and one control region. The overall base composition was A (28.8%), T (27.0%), C (26.7%), G (17.5%). Phylogenetics analyses of *N. Lutipinnis* and 29 closely related species found discrepancies between genetic relationships and taxonomic delineations, highlighting the need for further studies of phylogenetic and biogeographic relationships among the closely related taxa of the subfamily Pogonichthyinae.

*Notropis lutipinnis* (Jordan & Brayton, 1878), commonly known as the yellowfin shiner, is a cypriniform fish in the family *Leuciscidae* found in freshwater streams across the Southeastern United States from Alabama to North Carolina, in the Mobile, Tennessee, Apalachicola, Altamaha, Savannah, Edisto, and Santee River Drainages (Wood and Mayden 1992; Scott et al., 2009; Cashner et al. 2011). *N. lutipinnis* has a natural distribution in both Gulf and Atlantic coast drainage basins and is found in headwater streams, creeks, and small rivers. When present, *N. lutipinnis* is often among the most abundant fish species at a site, where it has unique ecological interactions with other species such as complex spawning aggregations and nest parasitism of the bluehead chub (*Nocomis leptocephalus*).

Genomic DNA was isolated from a caudal fin clip of a specimen collected from Turnpike Creek (35.15141, −84.26057), a tributary to the Flint River; the voucher is in the Georgia Museum of Natural History Tissue Collection (GMNHTC# 11921). DNA was isolated using the Puregene DNA extraction kit, and sequenced by Illumina MiSeq (250 bp paired-end reads) at the Georgia Genomics and Bioinformatics Core (formerly the Georgia Genomics Facility). Raw reads were trimmed and quality checked with Trim Galore (Krueger 2012). The Geneious Read Mapper algorithm (Kearse et al. 2012) in Geneious 8.1.9 was used to map reads to the mitochondrial genome of *Notropis chrosomus* (AP012108.1 ref). We found 99642 reads mapped to the *N. chrosomus* reference. The mapped reads were then used to create a *de novo* assembly with mean coverage of 1308.4 ± 204.9. No large structural variants were observed between *N. lutipinnis* and *N. chrosomus* mitochondrial genomes. Annotations were completed with Mitofish Annotator (Iwasaki et al. 2013). The complete, annotated mitochondrial sequence is accessible through GenBank under accession number MT333789.

The complete mitochondrial genome of *Notropis lutipinnis* (16,706 bp) consists of 13 protein coding genes, two rRNA genes, 22 tRNA genes, and the control region (D-loop), as expected for a vertebrate mitochondrial genome. The tRNA genes varied in length from 68 bp (*tRNA-Cys*) to 76 bp (*tRNA-Leu*). The overall base composition was A (28.8%) > T (27.0%) > C (26.7%) > G (17.5%). The percentage of GC (44.2%) was lower than AT. The start codon for all protein coding genes was ATG, with the exception of Cytochrome c Oxidase subunit I (COXI), which was GTG (Delarbre et al. 1997). Six protein coding genes use the stop codon TAA, two use the incomplete stop codon TA− and the remaining five use the incomplete stop codon T–. Presumably, these are cleaved at the base immediately following the partial stop codon during RNA processing, to keep the start codon of the subsequent gene intact, then converted to TAA stop codons upon poly-adenylation (Ojala et al. 1981; Clayton 2000). The heavy strand acts as the coding strand for the majority of protein coding genes and tRNAs, however one protein coding gene (ND6) and 8 of 22 tRNAs (*tRNA-Pro*, *tRNA-Glu*, *tRNA-Ser*, *tRNA-Tyr*, *tRNA-Cys*, *tRNA-Asn*, *tRNA-Ala*, *tRNA-Gln*) use the light strand as the coding strand.

Phylogenetic analyses were completed using the complete mitochondrial genome sequence of *N. lutipinnis*, 28 other species of the subfamily *Pogonichthyinae*, and *Phenacobius mirabilis* (another *Pogonichthyinae*) as an outgroup (Schönhuth et al. 2018). Sequences were aligned with MUSCLE (Edgar 2004) with the maximum number of iterations set to 8. Iteration 1 of the alignment used the kmer4_6 distance measure, while iteration 2 used pctid_kimura, all iterations used the UPGMB distance measure, pseudo tree rooting, the CLUSTALW sequence weighting scheme, half penalty for terminal gaps, spm objective score, anchor spacing 32, open gap score of −1, minimum length of 24, margin of 5, minimum column anchor score of 90, hydrophobicity multiplier of 1.2 and, window size of 5. The phylogenetic tree was built with RaxML (Stamatakis 2014) implemented in Geneious using the GTR + G + I model of nucleotide substitution, new rapid hill climbing as the tree search algorithm, and 1000 inferences of the original tree on distinct randomized maximum parsimony trees (Figure 1).

**Figure 1.**
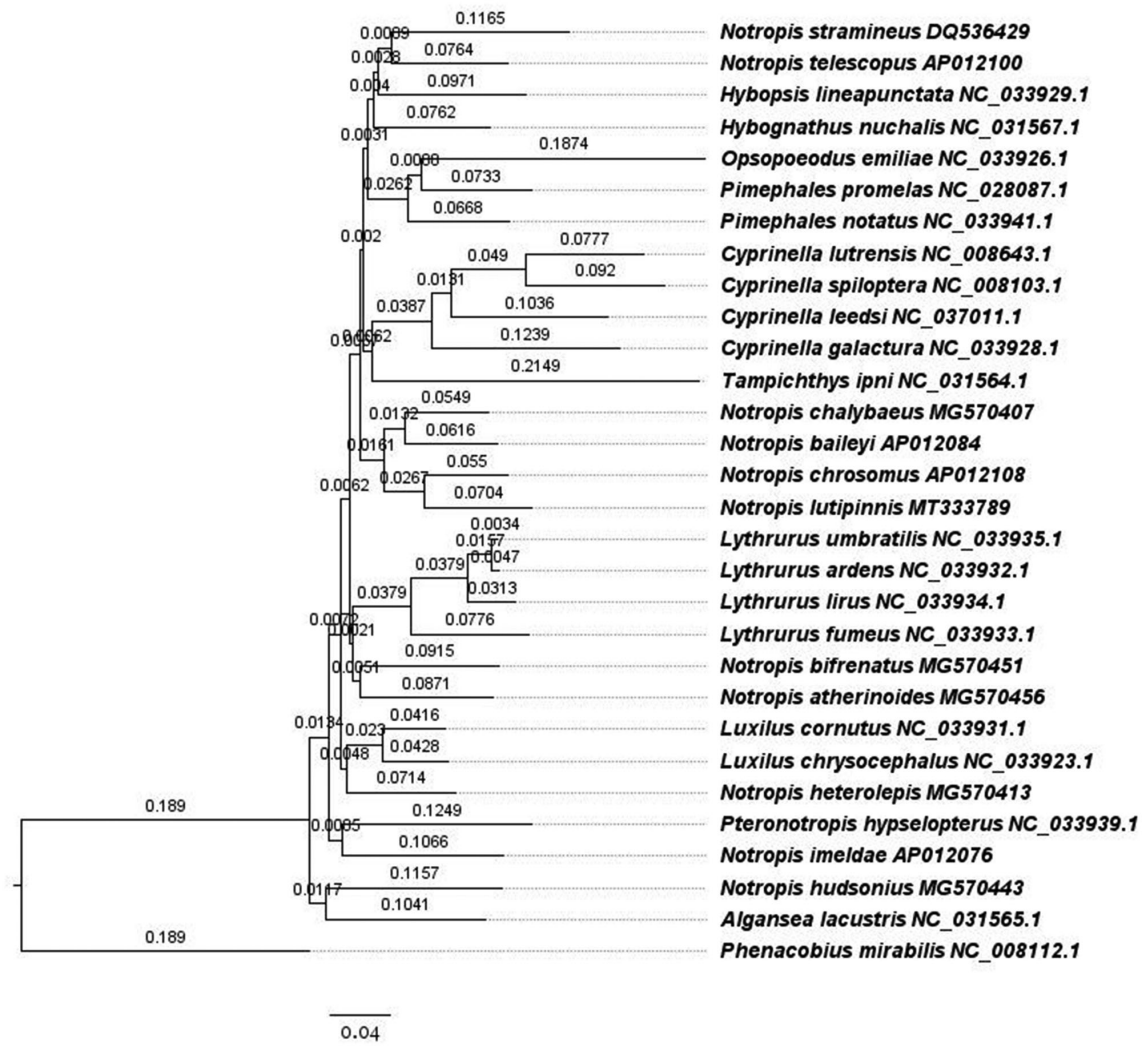
Phylogenetic tree of shiner mitochondrial genomes built with RAxML, branch labels are substitutions per site.

We found that mitochondrial markers indicate that the genus *Notropis* is polyphyletic, whose species are interspersed with other genera as part of a larger monophyletic clade within the subfamily Pogonichthyinae of the family Leuciscidae. Species of *Notropis* phylogenetically cluster with *Algansea*, *Cyprinella*, *Hybopsis*, *Hybognathus*, *Luxilus*, *Opsopoeodus*, *Lythrurus*, *Pimephales*, *Pteronotropis*, and *Tampichthes*. Similar results have been found in broader phylogenetic studies (Schönhuth et al. 2018). The combination of the phylogenetic relationships found here and in previous studies suggest detailed genetic studies of this group should be conducted to clarify taxonomic and phylogenetic relationships.

The availability of this complete mitochondrial genome will support future ecological and biogeographic studies within this widespread species – including questions about how fishes end up on opposite sides of the continental divide (Johnson 1907) and across the diverse lineage of *Notropis*.

## Data Availability

Data that support the findings of this study are openly available in Genbank with reference accession number MT333789 at https://www.ncbi.nlm.nih.gov/nuccore/MT333789.

## Geolocation information

The specimen was collected under the Georgia Department of Natural Resources Permit Number 29-WBH-12-129 issued to Byron J. Freeman, on August 6, 2012 by Mary C Freeman, and Rachel A Katz at Turnpike Creek, Flint River Drainage, Georgia, 33.152829-84.261203, Site: Turnpike Creek DS of Perkins Rd crossing. The specimen has been added to the Georgia Museum of Natural History’s Tissue Collection as GMNH 11921.
